# The possible mechanism of Hippophae fructus oil applied in tympanic membrane repair identified based on network pharmacology and molecular docking

**DOI:** 10.1002/jcla.24157

**Published:** 2021-12-03

**Authors:** Juntao Huang, Bing Mei Teh, Ziqian Xu, Zhechen Yuan, Chongchang Zhou, Yunbin Shi, Yi Shen

**Affiliations:** ^1^ Department of Otolaryngology Head and Neck Surgery Ningbo Medical Center (Ningbo Lihuili Hospital) The Affiliated Lihuili Hospital of Ningbo University Ningbo China; ^2^ School of Medicine Ningbo University Ningbo China; ^3^ Department of Ear Nose and Throat, Head and Neck Surgery Eastern Health Box Hill Victoria Australia; ^4^ Department of Otolaryngology, Head and Neck Surgery Monash Health Clayton Victoria Australia; ^5^ Faculty of Medicine, Nursing and Health Sciences Monash University Clayton Victoria Australia; ^6^ Department of Dermatology Shanghai General Hospital Shanghai Jiao Tong University School of Medicine Shanghai China

**Keywords:** Hippophae fructus, molecular docking, network pharmacology, tympanic membrane perforation, wound healing

## Abstract

**Objective:**

This study aimed to explore the mechanisms of Hippophae fructus oil (HFO) in the treatment of tympanic membrane (TM) perforation through network pharmacology‐based identification.

**Methods:**

The compounds and related targets of HFO were extracted from the TCMSP database, and disease information was obtained from the OMIM, GeneCards, PharmGkb, TTD, and DrugBank databases. A Venn diagram was generated to show the common targets of HFO and TM, and GO and KEGG analyses were performed to explore the potential biological processes and signaling pathways. The PPI network and core gene subnetwork were constructed using the STRING database and Cytoscape software. A molecular docking analysis was also conducted to simulate the combination of compounds and gene proteins.

**Results:**

A total of 33 compounds and their related targets were obtained from the TCMSP database. After screening the 393 TM‐related targets, 21 compounds and 22 gene proteins were selected to establish the network diagram. GO and KEGG enrichment analyses revealed that HFO may promote TM healing by influencing cellular oxidative stress and related signaling pathways. A critical subnetwork was obtained by analyzing the PPI network with nine core genes: CASP3, MMP2, IL1B, TP53, EGFR, CXCL8, ESR1, PTGS2, and IL6. In addition, a molecular docking analysis revealed that quercetin strongly binds the core proteins.

**Conclusion:**

According to the analysis, HFO can be utilized to repair perforations by influencing cellular oxidative stress. Quercetin is one of the active compounds that potentially plays an important role in TM regeneration by influencing 17 gene proteins.

## Graphical abstract

The mechanism of hippophae fructus oil in treatment of tympanic membrane perforations was explored by the technology of network pharmacology and molecular docking. Hippophae fructus oil may repair perforations by influencing the regulation of cellular oxidative stress, and the quercetin may be one of the possibly effective compounds in tympanic membrane repair. This study provided a novel idea to explore new drugs in tympanic membrane repair.

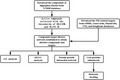

## 1 INTRODUCTION

Tympanic membrane (TM) perforation is a common finding in otology clinics that causes hearing loss and infections and consequently decreases the quality of human life.^1^, ^2^ Persistent TM perforation with infection, if left untreated, usually requires surgical interventions.^2^, ^3^ Patients who undergo tympanoplasty may suffer from pain and potential postoperative complications and might incur high medical costs.^3^, ^4^ For simple perforations without other middle ear disorders (e.g., cholesteatoma), an alternative to rupture closure that is less invasive and expensive is preferable to surgical intervention.^5^, ^6^, ^7^

Hippophae fructus oil (HFO), a traditional Chinese herb extracted from Hippophae fructus,^8^, ^9^, ^10^ is composed of vitamin C, amino acids, and essential trace elements (e.g., zinc), which can enhance the proliferation of TM epithelial stem cells, promote their migration to lesions and induce blood vessel dilatation.^9^, ^10^ During the past few decades, HFO has been applied for the clinical treatment of TM perforation with satisfactory outcomes.^8^ However, the biological mechanism of HFO therapy for TM regeneration remains unclear, and the main compounds of HFO that promote TM healing require further exploration.

The development of biological informatics engineering has made it possible to explore the mechanisms of Chinese herbs used for disease treatment. Network pharmacology combines pharmacokinetic and pharmacodynamic properties with the field of systems biology to study drugs, protein targets, and their pharmacological activities.^11^, ^12^ Hence, we explored the underlying mechanism of HFO in TM repair using the technologies of network pharmacology and molecular docking.

## 2 MATERIALS AND METHODS

### 2.1 Identification of active compounds of HFO and corresponding target genes

Detailed information on the compounds, targets and other secondary data related to HFO was obtained from the Traditional Chinese Medicine System Pharmacology Database (TCMSP, http://tcmspw.com/tcmsp.php) with the keywords Hippophae Fructus.^13^ Specifically, oral bioavailability (OB) and drug likeness (DL), which are the most important indicators for evaluating the characteristics of absorption, distribution, metabolism, and excretion (ADME), were used to filter the candidate active compounds based on the thresholds OB ≥ 30% and DL ≥ 0.18.^14^ For each active compound, we searched related target genes in the TCMSP database. An HFO gene set was then mapped using the UniProt database by limiting the species to “Homo sapiens” (https://www.uniprot.org/) to obtain gene symbol annotation.^15^

### 2.2 Obtainment of the target genes related to TM

The TM‐related targets were mined by searching the following five databases: Online Mendelian Inheritance in Man (OMIM; https://omim.org/) database,^16^ GeneCards (https://www.genecards.org/) database,^17^ PharmGkb (https://www.pharmgkb.org/) database,^18^ Therapeutic Target Database (TTD) (http://db.idrblab.net/ttd/),^19^ and DrugBank (https://www.drugbank.ca/) database.^20^ A TM‐related gene set was established by combining the search results.

### 2.3 Compound‐target pharmacology network and functional analysis

After preparing two sets of target lists for the HFO‐related genes and disease targets, a screening for drug‐disease crossover was performed. The common targets were selected, and a Venn diagram was generated using the Venn Diagram package of R software to visualize the intersection sets. The compound‐target‐disease network diagram was prepared using Cytoscape version 3.8.0 software to show the relationships among the TM, HFO, and gene targets.^21^

A functional enrichment analysis, including gene ontology (GO) analysis and Kyoto Encyclopedia of Genes and Genomes (KEGG) pathway analysis, was performed to elucidate the underlying mechanism through assessments of biological processes (BPs), cellular components (CCs), molecular functions (MFs), and key signaling pathways. The intersecting proteins were then evaluated by bioinformatics annotation using the “clusterProfiler” and “bioconductor” packages of R software. The significantly enriched GO and KEGG terms were identified, and *p*‐values < 0.05 and *q*‐values < 0.05 indicated a strong association with related BPs.^22^, ^23^

### 2.4 Protein–protein interaction network and critical subnetwork

For identification of the potential targets of HFO and the interactions between them, the Search Tool for the Retrieval of Interacting Genes/Proteins (STRING) (http://string‐db.org/) database^24^ was used to analyze the intersecting protein–protein interactions (PPIs) through the intersection set between HFO targets and TM‐related genes. After importing the PPI network into Cytoscape software, a critical subnetwork was investigated using the CytoNCA plugin.^25^ The values of betweenness, closeness, degree, eigenvector, and local average connectivity‐based method, as well as the network scores, were independently calculated, and the eligible genes with scores higher than the median value were selected to establish a critical subnetwork.^25^

### 2.5 Molecule docking technology

The compound with the largest number of critical genes related to TM was selected for further analysis based on molecular docking. The 2D structure of the molecular ligands was downloaded from the PubChem database (https://pubchem.ncbi.nlm.nih.gov/),^26^ and the 3D structure with minimum energy was calculated and exported using ChemBio3D software.^27^ Furthermore, the 3D structure of the receptor protein encoded by the selected gene was obtained from the UniProt database and downloaded from the RCSB PDB database (https://www.rcsb.org/).^28^

After preparing the files of the 3D structure, the receptor proteins were dehydrated, and the ligands were removed using PyMOL software.^29^ AutoDock Tools was used to modify the receptor protein by calculating the hydrogenation and charges of proteins. Moreover, the parameters of the receptor protein docking site were set to include the active pocket sites where small molecule ligands bind, and the molecular docking between the compound and receptor proteins was displayed using AutoDock Vina software.^30^

## 3 RESULTS

### 3.1 Active compounds and potential targets

**FIGURE 1:**
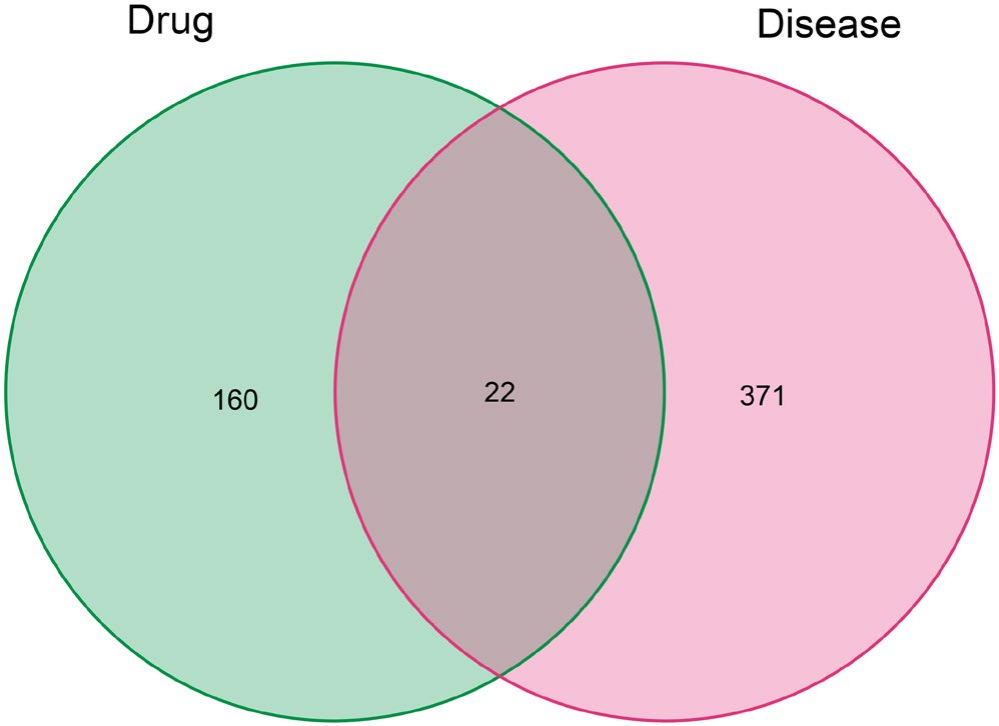
Venn diagram of gene intersections between HFO and TM perforation. HFO, Hippophae fructus oil; TM, tympanic membrane

Using the criteria DL ≥ 0.18 and OB ≥ 30%, 33 main and effective compounds were retrieved and selected (Table 1). A total of 182 compound‐related targets were annotated into a gene symbol set using the UniProt database. In addition, 393 TM‐related targets were extracted from the OMIM, GeneCards, PharmGkb, TTD, and DrugBank databases after removing duplicates. Moreover, an intersection of the compound‐target and disease‐related genes, which contained 22 target proteins, was obtained and is shown in Figure 1.

**TABLE 1:** Active compounds of hippophae fructus

Mol ID	Molecule name	OB (%)	DL
MOL001004	Pelargonidin	37.98831233	0.21204
MOL010211	14,15‐dimethyl‐alpha‐sitosterol	43.13700612	0.78478
MOL010212	14‐methyl‐alpha‐sitosterol	43.48505263	0.78028
MOL010227	Canthaxanthine	41.58914575	0.56161
MOL010228	Carotenoid	40.75960813	0.54932
MOL010230	ST5330591	48.07729226	0.84329
MOL010232	Cislycopene	45.51347546	0.54476
MOL010234	Delta‐Carotene	31.80094312	0.54639
MOL010240	Ergosta‐7‐en‐3‐beta‐ol	38.76055617	0.82626
MOL010241	Ergostenol	35.40870144	0.71393
MOL010247	(2R,6S,7aR)‐2‐[(1E,3E,5E,7E,9E,11E,13E,15E)‐16‐[(1R,4R)‐4‐hydroxy‐2,6,6‐trimethyl‐1‐cyclohex‐2‐enyl]‐1,5,10,14‐tetramethylhexadeca‐1,3,5,7,9,11,13,15‐octaenyl]‐2,4,4,7a‐tetramethyl‐6,7‐dihydro‐5H‐benzofuran‐6‐ol	57.88019692	0.52897
MOL010248	Gamma‐carotene	30.98275266	0.55342
MOL001979	LAN	42.11918897	0.74787
MOL010267	LYC	32.57391809	0.50916
MOL010283	ZINC03831331	47.60362222	0.65038
MOL001420	ZINC04073977	37.99618556	0.75755
MOL001494	Mandenol	41.99620045	0.19321
MOL001510	24‐epicampesterol	37.57681789	0.71413
MOL002268	Rhein	47.06520991	0.27678
MOL002588	(3S,5R,10S,13R,14R,17R)‐17‐[(1R)‐1,5‐dimethyl‐4‐methylenehexyl]‐4,4,10,13,14‐pentamethyl‐2,3,5,6,7,11,12,15,16,17‐decahydro‐1H‐cyclopenta[a]phenanthren‐3‐ol	42.36819868	0.76765
MOL002773	Beta‐carotene	37.18433337	0.58358
MOL000354	Isorhamnetin	49.60437705	0.306
MOL000358	Beta‐sitosterol	36.91390583	0.75123
MOL000359	Sitosterol	36.91390583	0.7512
MOL000422	Kaempferol	41.88224954	0.24066
MOL000433	FA	68.96043622	0.7057
MOL000449	Stigmasterol	43.82985158	0.75665
MOL000492	(+)‐catechin	54.82643405	0.24164
MOL005100	5,7‐dihydroxy‐2‐(3‐hydroxy‐4‐methoxyphenyl)chroman‐4‐one	47.73643694	0.27226
MOL006756	Schottenol	37.42312067	0.75067
MOL000073	Ent‐Epicatechin	48.95984114	0.24162
MOL000953	CLR	37.87389754	0.67677
MOL000098	Quercetin	46.43334812	0.27525

Abbreviations: OB, oral bioavailability; DL, drug likeness.

### 3.2 Network analysis of targets

A compound–disease–target interaction network with 43 nodes and 66 edges was visualized using Cytoscape software. The possible effective compounds of HFO related to TM perforation repair are the following: pelargonidin, 14‐methyl‐alpha‐sitosterol, ergosterol, LAN, ZINC04073977, mandenol, 24‐epicampesterol, rhein, beta‐carotene, isorhamnetin, beta‐sitosterol, sitosterol kaempferol, stigmasterol, (+)‐catechin, schottenol, ent‐epicatechin, CLR, quercetin, (3S,5R,10S,13R,14R,17R)‐17‐((1R)‐1,5‐dimethyl‐4‐methylenehexyl)‐4,4,10,13,14‐pentamethyl‐2,3,5,6,7,11,12,15,16,17‐decahydro‐1H‐cyclopenta(a)phenanthren‐3‐ol, and 5,7‐dihydroxy‐2‐(3‐hydroxy‐4‐methoxyphenyl)methoxy‐chroman‐4. Moreover, the 22 related gene proteins were PTGS1, PTGS2, PGR, NR3C1, MMP2, CASP3, CYP3A4, ESR1, MAP2, SLC6A2, CYP1B1, MMP3, EGFR, MMP9, IL6, TP53, RAF1, IL1B, CXCL8, HSPB1, NFE2L2, and SPP1.

**FIGURE 2:**
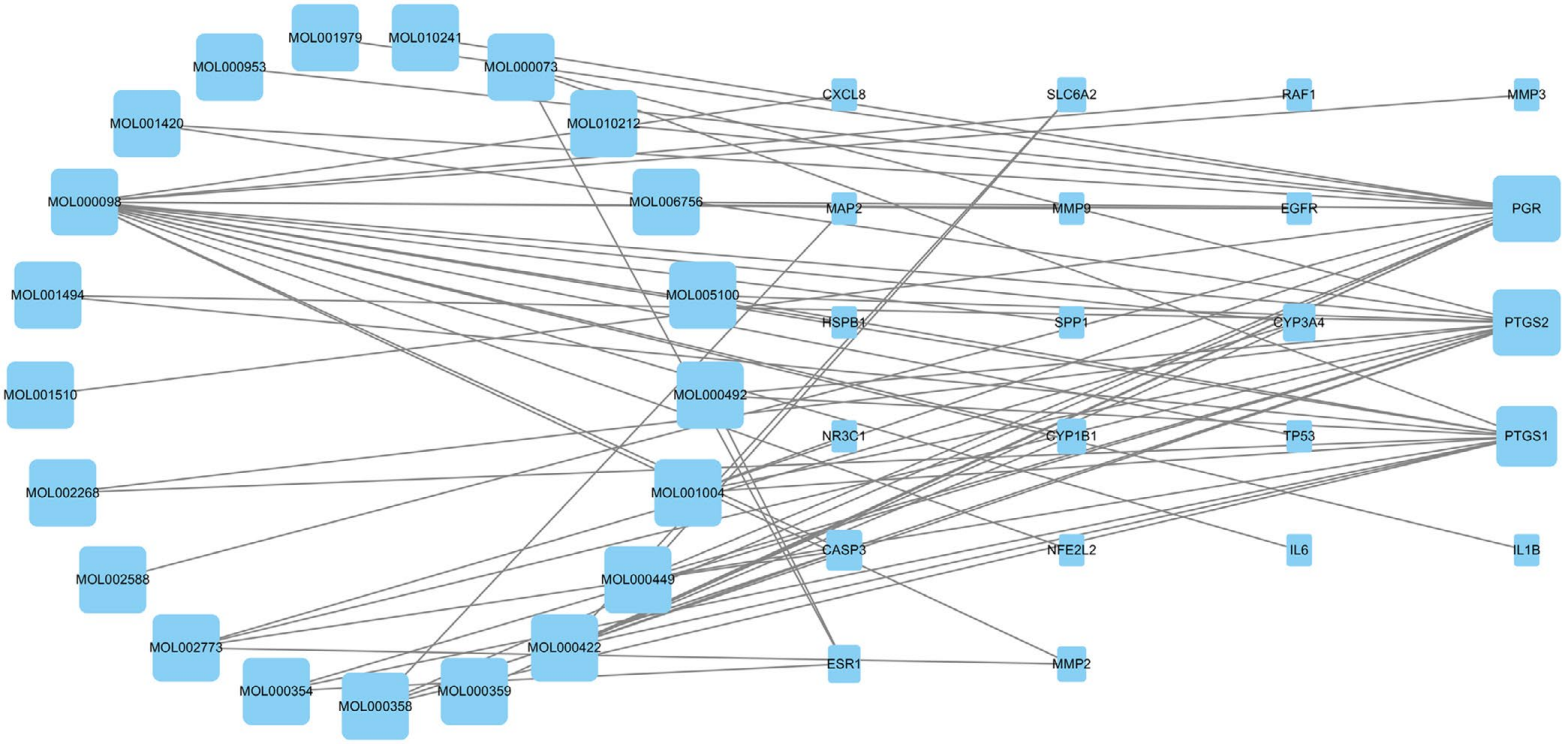
HFO‐compounds‐targets‐TM perforation network diagram. HFO, Hippophae fructus oil; TM, tympanic membrane

As shown in Figure 2, PTGS2 and PGR were identified as two genes that were most frequently targeted by the HFO ingredients, and both of these genes were targeted by 13 active compounds. Furthermore, quercetin may be one of the primary therapeutic compounds affecting 17 target proteins on the TM and may play a critical role in the healing process.

### 3.3 GO and KEGG enrichment analysis

**FIGURE 3:**
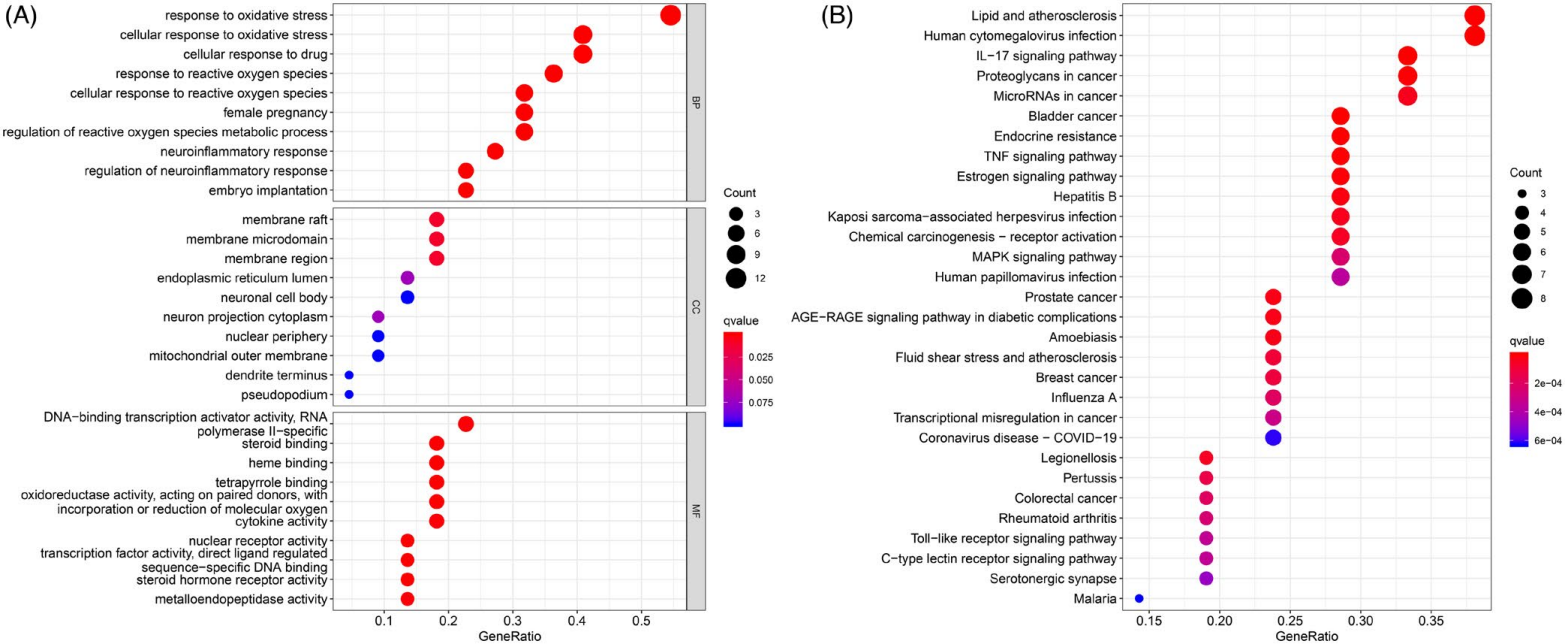
GO (A) and KEGG (B) enrichment analysis. GO, gene ontology; HEGG, Kyoto Encyclopedia of Genes and Genomes

The GO enrichment analysis revealed the underlying BPs, CCs, and MFs of the 22 target genes. Using the criteria *p*‐value < 0.05 and *q*‐value < 0.05, a total of 1208 significantly enriched GO terms were obtained. The top 10 terms are illustrated in Figure 3. The analysis of BPs revealed that the targets were enriched in different GO terms, and the predicted therapeutic targets were mainly associated with three BPs, namely response to oxidative stress, cellular response to oxidative stress, and cellular response to drugs; three CCs, namely membrane raft, membrane microdomain, and membrane region; and one MF, namely DNA‐binding transcription activator activity (RNA polymerase II−specific).

Using the filters *p*‐value < 0.05 and *q*‐value < 0.05, a KEGG enrichment analysis was performed to elucidate the related pathways. The results revealed that 103 potential signaling pathways were enriched, and the top 30 of these pathways are shown in Figure 3. The bubble plot demonstrates that these gene targets affected pathways related to infection and wound healing, such as the IL‐17 signaling pathway.

### 3.4 PPI diagram and core subnetwork

**FIGURE 4:**
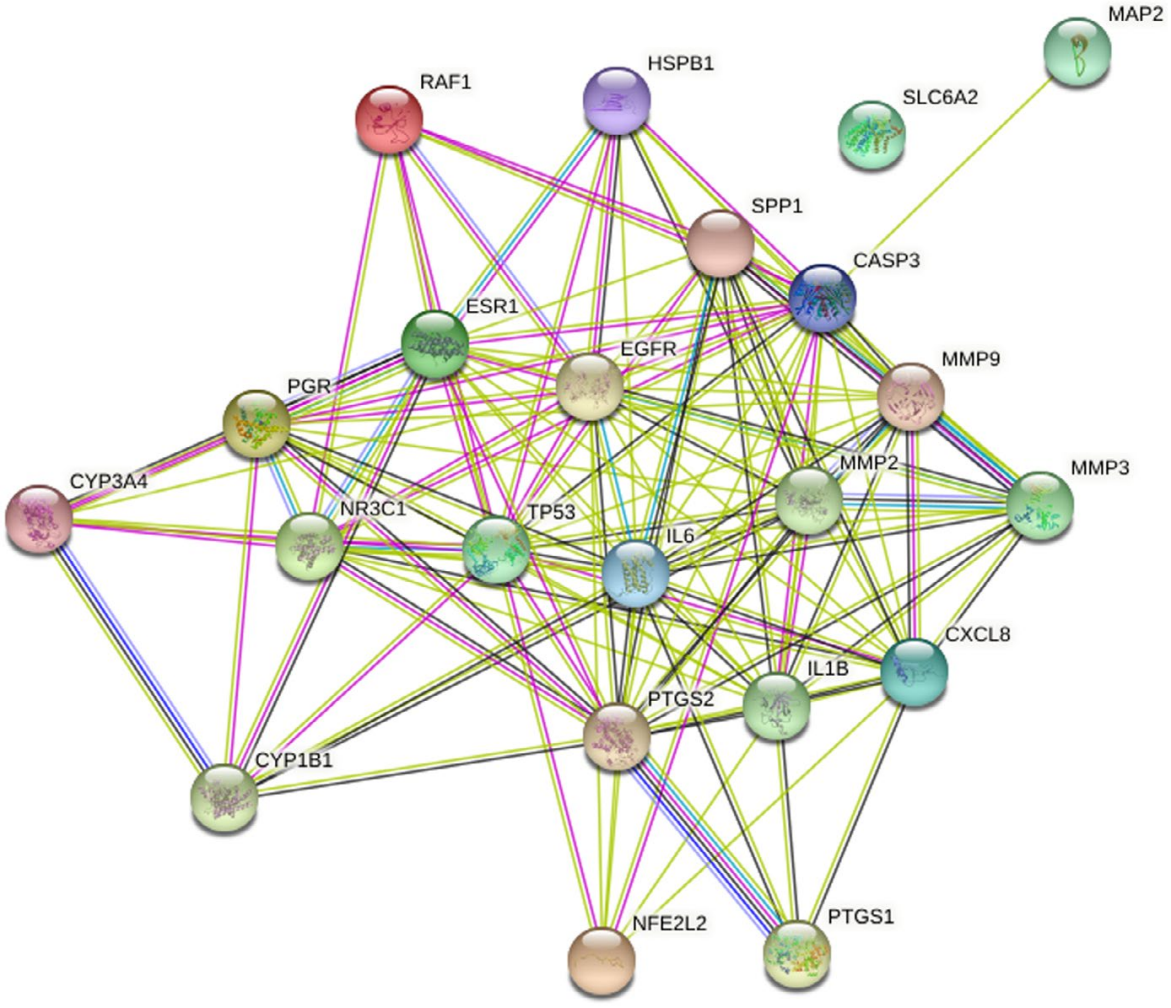
Protein–protein interactions diagram

**FIGURE 5:**
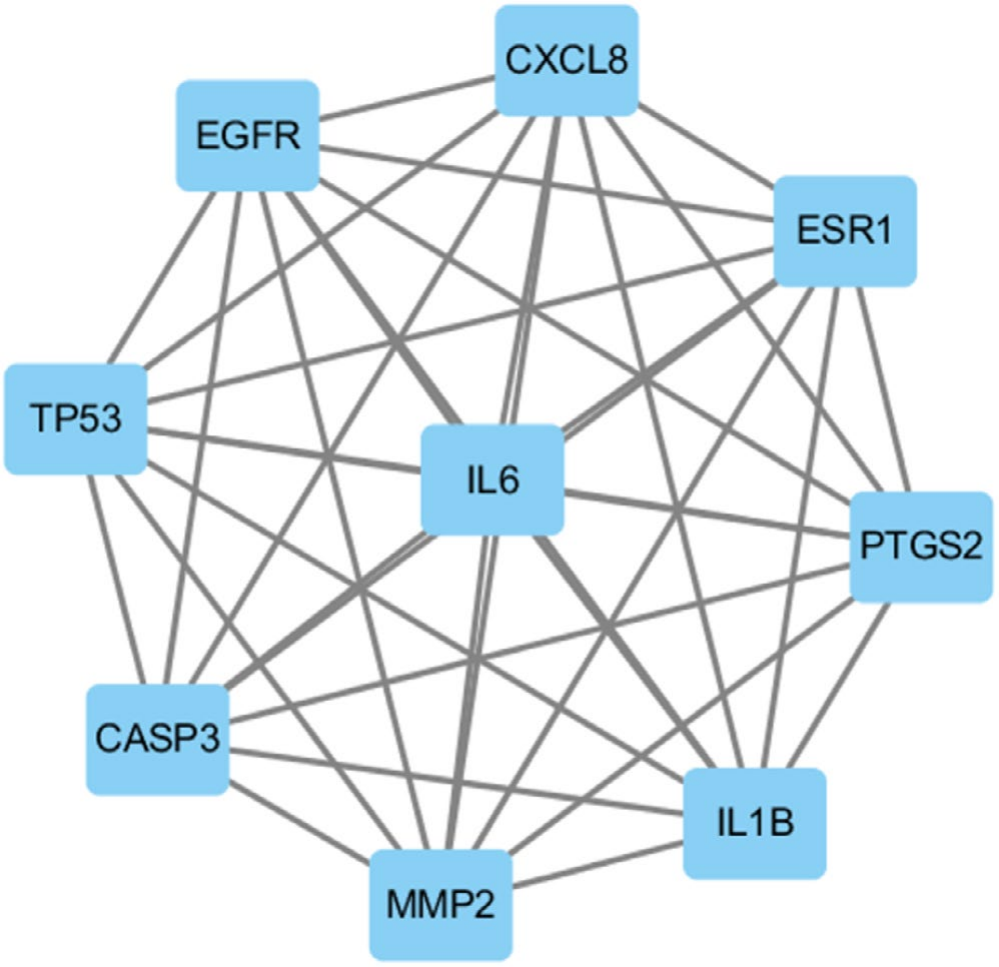
Core proteins subnetwork diagram

Twenty‐two overlapping genes associated with TM and HFO were inputted into the STRING database, and a PPI network diagram was established after selecting “Homo sapiens.” As shown in Figure 4, the network contained 22 nodes and 123 edges. In addition, while importing the results from the PPI network into Cytoscape and using the CytoNCA plugin, a further core subnetwork that contained nine nodes and 36 edges was obtained (Figure 5). These nine core gene targets included CASP3, MMP2, IL1B, TP53, EGFR, CXCL8, ESR1, PTGS2, and IL6. The results from the GO and KEGG analyses revealed that the core genes are involved in the cellular response to oxidative stress and play critical roles in signaling pathways. Detailed information on the compounds is summarized in Table 2.

**TABLE 2:** Core proteins and related active compounds

Targets	Compounds
CASP3	Beta‐carotene; beta‐sitosterol; kaempferol; quercetin
EGFR	Quercetin
MMP2	Beta‐carotene; quercetin
TP53	Quercetin
PTGS2	Pelargonidin; ZINC04073977; Mandenol; rhein; beta‐carotene; isorhamnetin; beta‐sitosterol; kaempferol; Stigmasterol; (+)‐catechin; 5,7‐dihydroxy‐2‐(3‐hydroxy‐4‐methoxyphenyl) chroman‐4‐one; ent‐Epicatechin; quercetin
IL1B	Quercetin
CXCL8	Quercetin
ESR1	Isorhamnetin; (+)‐catechin; ent‐Epicatechin
IL6	Quercetin

### 3.5 Molecular docking

**FIGURE 6:**
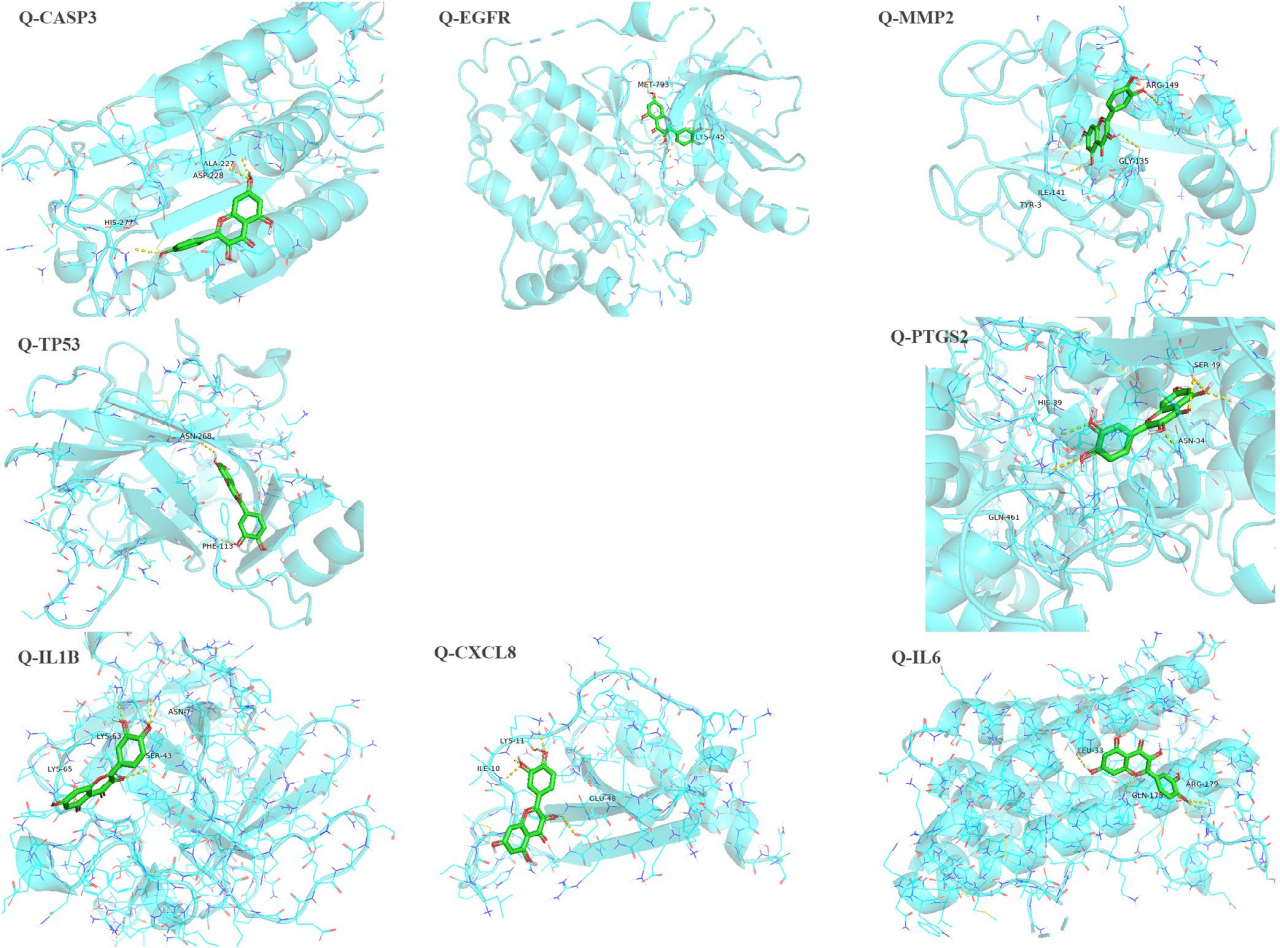
Visualized results for molecular docking

Referring to the results of the core gene network, we identified quercetin as a ligand of CASP3, MMP2, IL1B, TP53, EGFR, CXCL8, PTGS2, and IL6 protein receptors. A further calculation was then conducted to simulate the molecular docking of quercetin with these eight gene proteins, and the docking affinity values are listed in Table 3. A greater absolute value for the docking affinity indicates stronger binding ability between the active site of the protein receptor and the compound. The docking results indicate that quercetin can easily enter and bind the active pocket of the eight core target proteins, can form hydrogen bonds with the amino acid residues, and exhibits high binding affinity (Figure 6).

**TABLE 3:** Detailed results for molecular docking

Targets	PDB code	Affinity (kcal/mol)
CASP3	2DKO	−6.7
EGFR	2RGP	−8.7
MMP2	3AYU	−8.6
TP53	2PCX	−7.0
PTGS2	5KIR	−9.6
IL1B	5R7W	−7.2
CXCL8	3IL8	−5.9
IL6	1ALU	−6.9

## 4 DISCUSSION

Tympanic membrane perforation can lead to severe morbidity and disability.^2^, ^3^ With the development of tissue engineering, various bioactive materials (e.g., growth factors) have been used as a replacement to conventional tympanoplasty in the treatment of simple TM perforations with great success rates.^1^, ^2^, ^3^, ^4^, ^5^, ^6^, ^7^ However, these molecules have their own limitations, such as otorrhea caused by a high dosage of growth factors, and an ideal bioactive material has not yet been identified.^2^, ^7^ In traditional Chinese medicine, doctors use Chinese herbs to repair TM perforations, and these herbs have been reported to accelerate the wound healing process.^31^, ^32^, ^33^ Previous studies have suggested that HFO may be an effective, safe and convenient herb for promoting the healing of TM perforations.^8^ However, the underlying mechanism of HFO in TM repair remains unknown. Thus, in this study, we performed a network pharmacology‐based analysis to explore the mechanism of HFO in TM repair.

The results from the GO enrichment analysis revealed that the mechanism through which HFO promotes TM regeneration may be associated with cellular oxidative stress. During the process of tissue wound healing, the biological regulation of cellular oxidative stress generally plays a critical role in cell proliferation and migration.^34^, ^35^ The signaling pathways identified from the KEGG enrichment analysis also suggested that these gene proteins may contribute to this biological process. Although the healing process of the TM differs from that of other tissues and despite the lack of direct evidence regarding the relationship of TM healing with oxidative stress, previous studies have indicated that individuals with chronic otitis media (particularly those accompanied by middle ear cholesteatoma) exhibit higher levels of oxidative stress markers in both the discharge fluid and serum than normal controls.^36^, ^37^, ^38^, ^39^ This evidence may support our finding that HFO may promote TM healing by regulating biological processes related to cellular oxidative stress.

The PPI network diagram reflected the protein–protein relationships among the 22 overlapping gene proteins. Moreover, a further critical subnetwork indicated that the nine core gene proteins might play a major role in the biological process of TM regeneration in response to HFO. Among these nine proteins, the EGFR gene protein reportedly plays an important role in TM healing. EGFR consists of an extracellular ligand‐binding domain, a transmembrane region, and an internal tyrosine kinase domain. This protein is localized in the basal layer of the stratified squamous epithelium of the TM and is involved in the regulation of cell growth and differentiation.^40^ When the TM is perforated, growth factors (e.g., EGF) are secreted and bound by EGFR to induce the proliferation of epithelial and endothelial cells, fibroblasts, and keratinocytes.^40^ The activation of EGFR can induce the transcription of regulatory proteins that stimulate proliferation and induce angiogenesis and also enhances the proliferation, adhesion, and migratory ability of TM cells.^41^ Moreover, animal experiments suggest that the inhibition of EGFR may prolong the duration of TM regeneration due to its synergistic effects with growth factors.^42^

In addition to the EGFR protein, other gene proteins also play important roles in the TM healing process. Among these core proteins, IL1B and IL6 participate in the process of histological damage and inflammation, as demonstrated with acute animal models.^43^, ^44^ The activity of MMP2 is also hypothesized to be involved in destruction of the TM fibrous layer and the prognosis of otitis media.^45^, ^46^

According to our analysis, 21 ingredients of HFO may contribute to the regeneration of the TM. Among these ingredients, quercetin can be considered the most likely active compound of HFO that promotes TM healing because it influences 17 gene proteins. Although no direct evidence shows the effectiveness of quercetin in TM regeneration, studies have revealed healing benefits from the application of quercetin to a wound both in vivo and in vitro.^47^ Reportedly, quercetin has great antioxidant and anti‐inflammatory properties and can increase the wound contraction rate and protect tissues from oxidative damage.^48^, ^49^, ^50^ According to our analysis, quercetin can act on EGFR, which is associated with the cellular proliferation of epithelial and endothelial cells, fibroblasts, and keratinocytes. When HFO is applied to perforated TMs, quercetin is potentially bound by EGFR to induce proliferation and migration. In addition to the biological effects of quercetin on wound healing, other effective compounds of HFO also display potential efficacy in TM healing. For instance, beta‐sitosterol reportedly produces the rapid re‐epithelialization of wounds and can exert a synergistic effect on quercetin.^51^ In addition, as one of the trace elements with a high HFO content, zinc increases inflammatory cytokines, oxidative stress production, granulation, and re‐epithelization via the regulation of matrix metalloproteinases and its association with tripartite motif family proteins during membrane repair and wound healing.^52^, ^53^, ^54^

As a nonsurgical method for TM perforation treatment, HFO therapy can be easily administered in clinics. Gao et al utilized a sterile cotton patch with HFO to cover TM perforations. Similar to topical growth factor therapy, this treatment of traumatic perforations with HFO also does not require hospitalization, which results in less inconvenience and lower medical costs for the patients.^8^ Moreover, HFO displays a variety of advantages in addition to healing benefits and is less invasive.^8^, ^9^, ^10^, ^31^, ^32^ As an extract from berries and seeds of traditional medicinal plants, HFO is extensively available from many sources. In addition, HFO is easily prepared and does not require cold storage, which may significantly reduce the associated costs. In addition, this treatment has the ability to prevent ototoxicity and infection, which is crucial for TM healing and auditory reconstruction.^55^

Although HFO shows great healing potential for TM perforations, the number of studies on the clinical application of HFO is not as high as that of studies that investigated other bioactive materials, which may be due to its complex composition and unknown mechanism related to the TM.^9^, ^10^ Therefore, further studies, if possible, should not only concentrate on exploring the underlying mechanism but also focus on a more detailed assessment of its clinical effectiveness. In addition to the healing and hearing results of HFO applied to acute or chronic perforations, the most effective dosage, duration and frequency of the application, and the patching materials have not yet been fully investigated.^1^, ^4^

In addition to exploring the mechanism and efficacy of HFO in further studies, investigations should assess whether the ingredients of this mixture can also be applied for TM regeneration based on our analysis. Quercetin, for instance, has been utilized as a medicine‐loaded vehicle for topical delivery in wound healing according to previous studies.^47^, ^48^, ^49^, ^50^ The ability to quench reactive oxygen species and mitigate the inflammatory process is essential in wound healing after topical application.^49^, ^50^ Considering these advantages, the utilization of quercetin has led to the novel notion of using herb monomers combined with other bioactive materials and has resulted in the identification of promising topical prodrugs.

In this study, we employed network pharmacology to elucidate the multitarget effects of HFO on TM perforations. Additionally, we discussed several potential herb monomers for TM perforation treatment, which could contribute to the development of new therapeutic strategies. However, the possible effects of HFO revealed from the network analysis require further verification by in vitro biological experiments. This analysis provides theoretical evidence regarding the mechanism of HFO in TM regeneration that can be further explored in future studies.

## 5 CONCLUSION

According to the analysis, HFO can be utilized to effectively repair TM perforations by influencing the regulation of cellular oxidative stress. Quercetin is one of the active compounds of HFP that potentially plays an important role in TM regeneration by influencing 17 gene proteins. This pharmacology‐based approach helps elucidate the underlying mechanisms of HFO in repairing TM perforations, but further experimental exploration is needed for verification of these mechanisms.

## CONFLICT OF INTEREST

None.

## AUTHOR CONTRIBUTIONS

All persons designated as the authors have participated sufficiently in the work to take public responsibility for the content of the manuscript. All the authors ensured that they all gave substantial contributions.

## Data Availability

The data that support the findings of this study are openly available from TCMSP, GeneCardS, OMIM, TTD, PharmGkb, and DrugBank belong to public databases.
